# Social Determinants of Health and Cancer Prevention Guideline Behaviors

**DOI:** 10.1001/jamanetworkopen.2025.42330

**Published:** 2025-11-07

**Authors:** Kathryn E. Chiang, Heather M. Padilla, Tamora A. Callands, Jessica L. Muilenburg, Sicha Chantaprasopsuk, Lauren C. Bates-Fraser, Valeria Elahy, Alpa V. Patel, Erika Rees-Punia

**Affiliations:** 1 Department of Population Science, American Cancer Society, Atlanta, Georgia; 2 Department of Health Promotion and Behavior, College of Public Health, University of Georgia, Athens; 3 Winship Cancer Institute, Emory University, Atlanta, Georgia

## Abstract

**Importance:**

The American Cancer Society (ACS) Guideline Score captures collective adherence to health behavior recommendations, including a healthy diet, physical activity (PA), alcohol intake, and body mass index (BMI). Exploring the role of social determinants of health (SDoH) in health behavior engagement may present opportunities for improving health equity.

**Objective:**

To identify SDoH associated with co-occurring health behaviors captured by the ACS Guideline Score in a large US cohort.

**Design, Setting, and Participants:**

This cross-sectional study included adults in the Cancer Prevention Study-3 who enrolled between 2006 and 2013 at ACS community events across 35 US states, the District of Columbia, and Puerto Rico using data from comprehensive follow-up surveys administered in 2015. Data were analyzed from June to September 2024.

**Exposures:**

Self-reported race and ethnicity, marital status, education, household income, secondhand smoke exposure, and work status were examined. Rural-Urban Commuting Area codes and food desert status were classified according to US Department of Agriculture definitions.

**Main Outcomes and Measures:**

Co-occurring health behaviors were measured using a 0-to-8–point score quantifying adherence to the 2020 ACS Guidelines for Diet and PA for Cancer Prevention on diet, alcohol, healthy BMI maintenance, and PA. Scores of 8 represent complete adherence. Ordinal logistic regression models were used to cross-sectionally assess SDoH factors associated with co-occurring health behaviors measured by ACS Guideline Scores.

**Results:**

Of 142 085 participants (mean [SD] age, 52.0 [9.6] years; 111 694 women [78.6%]), 2415 identified as Asian, Native Hawaiian, or Pacific Islander (1.7%), 3267 identified as Black (2.3%), 7814 identified as Latino (5.5%), 126 739 identified as non-Hispanic White (89.2%), and 1989 identified as another racial and/or ethnic group (1.4%). The mean (SD) ACS Guideline Score was 4.6 (1.7). Compared with White participants, Asian, Native Hawaiian, and Pacific Islander participants had increased odds of higher ACS Guideline Scores (odds ratio [OR], 1.99; 95% CI, 1.86-2.14). Holding a graduate degree was associated with a 33% higher likelihood of a higher ACS Guideline Score (OR, 1.33; 95% CI, 1.30-1.36) compared with college graduates. Compared with working full-time, working part-time (OR, 1.62; 95% CI, 1.57-1.66) or being retired (OR, 1.26; 95% CI, 1.22-1.30) was associated with a higher score.

**Conclusions and Relevance:**

In this cross-sectional study, multiple SDoH factors associated with co-occurring health behaviors for diet, PA, BMI, and alcohol consumption were identified. These findings may aid in identifying populations most vulnerable to poor health behaviors, guiding future approaches for advancing health equity.

## Key Points

**Question:**

Are social, economic, and geographic factors associated with co-occurring health behaviors measured by the American Cancer Society Guideline Score for Diet and Physical Activity for Cancer Prevention?

**Findings:**

In this cross-sectional study of 142 085 US adults, participants who identified as Asian, female, had higher socioeconomic statuses, resided in non–food desert metropolitan areas, had no secondhand smoke exposure, or were retired were more likely to engage in healthy co-occurring behaviors, including diet, physical activity, limited alcohol consumption, and a healthy body weight.

**Meaning:**

These findings suggest that demographic, socioeconomic, and geographical factors are associated with health behaviors and present opportunities for improving health equity.

## Teaser

This cross-sectional study investigates the association between social determinants of health and health behaviors measured by the American Cancer Society Guideline for Diet and Physical Activity.

## Introduction

Nearly 50% of adverse health outcomes are accelerated by social determinants of health (SDoH), the social and environmental factors that influence an individual’s living, working, growing, and aging conditions.^1^ The role of SDoH on various health outcomes may be mediated by individual health behaviors, including but not limited to diet, physical activity (PA), alcohol consumption, and weight management.^1,2^ For example, race and ethnicity are associated with varying dietary patterns,^3^ and neighborhood environments that promote access to safe walking paths, parks, and grocery stores are associated with healthy behaviors such as PA, healthy eating habits, and community connection,^2^ but associations with other aspects of SDoH (eg, marital status or secondhand smoke exposure) and other co-occurring health behaviors are still unclear. More robust investigations into the associations between multiple SDoH and health behaviors are necessary.^4^

Health behaviors are important for disease prevention and historically have been investigated for their individual effects, but evolving evidence suggests greater successes among interventions targeting multiple health behaviors.^5,6^ Additionally, the co-occurrence of poor health behaviors is associated with a higher risk of both mortality and morbidity compared with the sum of individual impacts.^7,8,9^ This interrelationship among health behaviors emphasizes the need to better understand how health behaviors co-occur and contribute together to health outcomes. Despite this, a vast majority of literature evaluates the impact of health behaviors individually, rather than as a collective.

The 2020 American Cancer Society (ACS) Guideline for Diet and Physical Activity for Cancer Prevention provides lifestyle recommendations for diet, alcohol intake, body mass index (BMI; calculated as weight in kilograms divided by height in meters squared), and physical activity.^10^ Adherence to the ACS Guideline may serve as a combined, aggregate measure for co-occurring diet, PA, alcohol, and weight management behaviors. Numerous studies have demonstrated that adherence to the ACS Guideline is associated with a reduced risk of all-cause, cancer, and cardiovascular disease mortality.^11,12,13^ Furthermore, associations between SDoH and diet quality have been demonstrated, and specifically suggest that White individuals with limited income, Black individuals, people with low educational attainment, and individuals residing in a food desert or rural area have considerably poorer diet quality.^3^ However, the associations between SDoH and other components of the ACS Guideline Score, including the score as a whole, is unclear.

Adherence to the 2020 ACS Guideline as a measure for capturing co-occurring health behaviors may provide a better understanding of the associations with SDoH. This cross-sectional study sought to identify SDoH associated with co-occurring health behaviors, including diet, PA, alcohol consumption, and BMI, in a large, diverse cohort of US adults.

## Methods

### Study Population

This cross-sectional study used data from the Cancer Prevention Study-3 (CPS-3), a prospective cohort study of cancer incidence and mortality initiated by ACS that included participants recruited from 35 states, the District of Columbia, and Puerto Rico.^14^ CPS-3 was approved by the institutional review board at Emory University. This study follows the Strengthening the Reporting of Observational Studies in Epidemiology (STROBE) reporting guideline, and all participants provided written informed consent.

In CPS-3, over 304 000 participants aged 30 to 65 years without a history of cancer, except basal or squamous cell skin cancer, were enrolled at various community events between 2006 and 2013. At enrollment, participants provided blood samples and detailed family and lifestyle histories followed by the completion of a baseline survey at home. Repeat surveys were issued triennially to update exposure information. Due to space restrictions and participant burden, dietary assessment was not comprehensively collected until the first follow-up survey in 2015.^14^ A total of 186 638 participants returned the 2015 follow-up survey, with 177 345 participants (69.9% of the active cohort) completing the CPS-3 Food Frequency Questionnaire.^3,14^ Further detailed descriptions regarding participant characteristics, cohort study design, and recruitment are described elsewhere.^14^

### Exclusions

Individuals were excluded from this analysis if the following information was missing on the 2015 survey: physical activity (930 individuals), alcohol use (197 individuals), the full Food Frequency Questionnaire (9293 individuals), or race and/or ethnicity (1446 individuals). Participants categorized as underweight (BMI <18.5) were also excluded as it is not scored in the ACS Guideline Score, as were participants missing BMI data (3719 individuals) or with incomplete diet reporting (17 200 individuals), described in detail elsewhere.^15^

### Outcome: ACS Guideline Score

An a priori score was developed to quantify lifestyle behaviors consistent with adherence to the 2020 ACS Guideline on Diet and Physical Activity for Cancer Prevention^10^ and an earlier score using the 2006 ACS Guideline.^11^ Each of the 4 guideline components, including BMI, PA, alcohol intake, and diet, was weighted equally and scored from 0 to 2, with 2 indicating full adherence to recommendations and 0 signifying recommendations are not met (eTable 1 in Supplement 1).

Height and weight reported on the 2006 to 2013 baseline survey and weight from the 2015 follow-up survey were used to calculate BMI. Those with a normal weight BMI (18.5 to <24.9) at both time points received the highest score of 2; those with a BMI greater than or equal to 30 (ie, obese) at 1 or both time points, received the lowest score of 0; a score of 1 was given to all other BMI combinations. Physical activity was measured using self-reported minutes of moderate to vigorous PA (eg, walking, biking, swimming, tennis, and other aerobic activities) per week that were converted into metabolic equivalent (MET)-hours per week, with 15 or more receiving a score of 2, indicating recommendations were met or exceeded. MET-hours per week ranging from 7.5 to less than 15 earned a score of 1, and MET-hours per week below 7.5 received a 0. For alcohol, the least favorable score of 0 was awarded to those who consume more than 1 drink per day for female participants and more than 2 drinks per day for male participants; 1 was given to those who drank less than or equal to 1 drink per day for female participants and less than or equal to 2 drinks per day for male participants; nondrinking participants received 2.

Survey-specific and sex-specific quartiles were created for intake and varieties of fruits and vegetables, intakes of whole grains, red and processed meats, sugar-sweetened beverages, and highly processed food and refined grains. Higher scores were associated with those in the higher quartile of intakes and varieties for fruits and vegetables (0-3 points) and whole grain intake (0-3 points). Highest red and processed meat, sugar-sweetened beverages, and highly processed food and refined grain intake earned the lowest scores (combined and totaling to 3-0 points). The 4 dietary subscores were then summed to create a diet score ranging from 0 to 12, with 12 indicating the highest diet quality (eTable 2 in Supplement 1). To ensure diet received the same weight as the other 3 factors in the ACS Guideline, diet scores were rescaled on a 0-to-2–point scale based on approximate tertile distributions within the study population.

Total ACS Guideline Scores were then created by summing each category, with overall scores ranging from 0, denoting none of the recommendations were met, to 8, denoting full adherence to the recommendations. ACS Guideline Scores in the tail distributions (0, 1, and 2 for lowest scores; 7-8 for highest scores) were collapsed because of small numbers.

### Exposure Variables: SDoH

The World Health Organization’s Commission on Social Determinants of Health conceptual framework^1^ and Healthy People 2030^16^ domains for SDoH guided the selection of social, economic, and geographical factors measured in this study. Demographics (ie, age, gender, race and ethnicity, and marital status), economic stability (work status and income), neighborhood and environment factors (ie, urban vs rural residence, residing in a food desert, and secondhand smoke [SHS] exposure), and education level were included as exposure variables. Participants self-reported race and ethnicity separately at enrollment, with groups categorized as Asian, Native Hawaiian, or Pacific Islander; Black (non-Latino); Latino (all races); White (non-Latino), and other (ie, American Indian, Alaska Native, and a write-in race option). Data on race and ethnicity are included in this study because of the known associations between race, ethnitcity, and individual health behaviors.^3,40^ Participants were asked about SHS exposure, smoking status, gross annual household income, and current work status on the 2015 survey. Rural-Urban Commuting Area (RUCA) codes (metropolitan, micropolitan, small town, and rural) were classified from 2010 census and 2006 to 2010 American Community Survey data.^17^ Residence in a food desert was assessed using 2015 geocoded addresses and was categorized as living in a food desert or not on the basis of the US Department of Agriculture Food Access Research Atlas database.^18^

Joint exposure variables, selected a priori, were created to assess the intersectionality among various SDoH and Guideline scores. Joint variables containing the RUCA variable collapsed small town and rural due to sparsity within these categories.

### Statistical Analysis

Ordinal logistic regression models were used to cross-sectionally assess SDoH factors associated with co-occurring health behaviors measured by ACS Guideline Scores. Model 1 adjusted for age, sex, and energy intake; model 2 mutually adjusted for other SDoH (ie, included all main exposures). The proportional odds assumption was evaluated for all ordinal regression models using diagnostic plots and analysis of variance tests; no violations were detected. Seven separate 2-way interaction models were used to explore the association of 2 aspects of SDoH on ACS Guideline Scores. All interaction models were evaluated on the multiplicative scale using ordinal logistic regression, and statistical significance of interactions was evaluated using type III Wald analysis of deviance tests. The outcome reference group in all models was set at an ACS Guideline Score of 0 to 2; therefore, all results describe the odds of having a higher score over the lowest score. Two-tailed *P* < .05 was considered statistically significant. All analyses were conducted in R Studio Pro 2024.04.1 running R statistical software version 4.4.0 (R Project for Statistical Computing).

## Results

A total of 142 085 participants were included in the final analytical cohort, with a mean (SD) age of 52.0 (9.6) years; 111 694 were women (78.6%) and 30 391 were men (21.4%) (Table 1). Only 2680 women (2.4%) and 486 men (1.6%) received a score of 8, indicating recommendations were fully met. The mean (SD) ACS Guideline Score was 4.6 (1.7). In total, 2415 identified as Asian, Native Hawaiian, or Pacific Islander (1.7%); 3267 identified as Black (2.3%); 7814 identified as Latino (5.5%); 126 739 participants identified as White (89.2%); and 1989 identified as another racial and/or ethnic group (1.4%) (Table 1).

**Table 1.** Participant Characteristics According to American Cancer Society Guideline Scores Among Aging Adults in the Cancer Prevention Study-3

Characteristic	Participants, No. (%)					
	Score 0-2 (n = 18 744)	Score 3 (n = 20 567)	Score 4 (n = 27 205)	Score 5 (n = 29 808)	Score 6 (n = 25 242)	Score 7-8 (n = 20 519)
Age, mean (SD), y	51 (9)	52 (10)	52 (10)	52 (10)	53 (10)	53 (10)
Sex						
Female	15 664 (83.6)	16 311 (79.3)	20 648 (75.9)	22 508 (75.5)	19 513 (77.3)	17 050 (83.1)
Male	3080 (16.4)	4256 (20.7)	6557 (24.1)	7300 (24.5)	5729 (22.7)	3469 (16.9)
Race and ethnicity						
Asian, Native Hawaiian, or Pacific Islander	125 (0.7)	223 (1.1)	405 (1.5)	513 (1.7)	525 (2.1)	555 (2.7)
Black	617 (3.3)	546 (2.7)	650 (2.4)	622 (2.1)	480 (1.9)	317 (1.5)
Latino	1115 (5.9)	1199 (5.8)	1510 (5.6)	1547 (5.2)	1350 (5.3)	1046 (5.1)
White	16 640 (88.8)	18 271 (88.8)	24 228 (89.1)	26 716 (89.6)	22 576 (89.4)	18 357 (89.5)
Other^a^	247 (1.3)	328 (1.6)	412 (1.5)	410 (1.4)	311 (1.2)	244 (1.2)
Annual household income, $						
<50 000	4013 (22.4)	3732 (18.1)	4360 (16.0)	4156 (13.9)	3107 (12.3)	2350 (11.5)
50 000 to <75 000	3895 (20.4)	3997 (19.4)	5015 (18.4)	5092 (17.1)	3925 (15.5)	3099 (15.1)
75 000 to <100 000	3518 (18.8)	3799 (18.5)	4937 (18.1)	5098 (17.1)	4276 (16.9)	3330 (16.2)
100 000 to <125 000	2903 (15.5)	3277 (15.9)	4422 (16.3)	4819 (16.2)	4099 (16.2)	3277 (16.0)
≥125 000	4208 (22.4)	5452 (26.5)	8030 (29.5)	10 164 (34.1)	9372 (37.1)	8051 (39.2)
Unknown or missing	207 (1.1)	310 (1.5)	441 (1.6)	479 (1.6)	463 (1.8)	412 (2.0)
Education level						
High school or less	2052 (10.9)	1972 (9.6)	2211 (8.1)	1932 (6.5)	1268 (5.0)	728 (3.5)
Some college or 2-y degree	6796 (36.3)	6635 (32.3)	7894 (29.0)	7526 (25.2)	5490 (21.7)	3676 (17.9)
College graduate	5919 (31.6)	6801 (33.1)	9389 (34.5)	10 605 (35.6)	9235 (36.6)	7495 (36.5)
Graduate degree	3910 (20.9)	5095 (24.8)	7608 (28.0)	9645 (32.4)	9165 (36.3)	8575 (41.8)
Unknown or missing	67 (0.4)	64 (0.3)	103 (0.4)	100 (0.3)	84 (0.3)	45 (0.2 )
Rural-urban commuting area						
Metropolitan	14 404 (76.8)	16 228 (78.9)	21 677 (79.7)	24 437 (82.0)	21 195 (84.0)	17 527 (85.4)
Micropolitan	1969 (10.5)	1960 (9.5)	2422 (8.9)	2461 (8.3)	1793 (7.1)	1339 (6.5)
Small town	609 (3.2)	595 (2.9)	816 (3.0)	728 (2.4)	564 (2.2)	364 (1.8)
Rural	388 (2.1)	375 (1.8)	524 (1.9)	425 (1.4)	308 (1.2)	224 (1.1)
Unknown or missing	1374 (7.3)	1409 (6.9)	1766 (6.5)	1757 (5.9)	1382 (5.5)	1065 (5.2)
Residing in a food desert						
No	15 749 (84.0)	17 626 (85.7)	23 548 (86.6)	26 205 (88.9)	22 435 (88.9)	18 434 (89.8)
Yes	1615 (8.6)	1528 (7.4)	1887 (6.9)	1838 (6.2)	1422 (5.6)	1020 (5.0)
Unknown or missing	1380 (7.4)	1413 (6.9)	1770 (6.5)	1765 (5.9)	1385 (5.5)	1065 (5.2)
Work status						
Full time	13 859 (73.9)	14 651 (71.2)	19 083 (70.1)	20 237 (67.9)	16 344 (64.7)	12 247 (59.7)
Part time	1626 (8.7)	1930 (9.4)	2738 (10.1)	3497 (11.7)	3388 (13.4)	3513 (17.1)
Retired	1687 (9.0)	2253 (11.6)	3150 (11.6)	3635 (12.2)	3292 (13.0)	2766 (13.5)
Other	1247 (6.7)	1355 (6.6)	1712 (6.3)	1918 (6.4)	1729 (6.8)	1543 (7.5)
Unknown or missing	325 (1.7)	378 (1.8)	522 (1.9)	521 (1.7)	489 (1.9)	450 (2.2)
Marital status						
Married or living with partner	13 514 (72.1)	15 298 (74.4)	20 643 (75.9)	22 995 (77.1)	19 806 (78.5)	16 104 (78.5)
Never married	1835 (9.8)	1677 (8.2)	2100 (7.7)	2107 (7.1)	1576 (6.2)	1412 (6.9)
Divorced, separated, or widowed	3180 (17.0)	3324 (16.2)	4109 (15.1)	4291 (14.4)	3537 (14.0)	2817 (13.7)
Unknown or missing	215 (1.1)	268 (1.3)	353 (1.3)	415 (1.4)	323 (1.3)	186 (0.9)
Secondhand smoke exposure						
Never	10 831 (57.8)	12 577 (61.2)	17 259 (63.4)	20 117 (67.5)	18 149 (71.9)	15 803 (77.0)
Any	7832 (41.8)	7883 (38.3)	9810 (36.1)	9559 (32.1)	6976 (27.6)	4625 (22.5)
Unknown or missing	81 (0.4)	107 (0.5)	136 (0.5)	132 (0.4)	117 (0.5)	91 (0.4)
Smoking status						
Never	11 980 (63.9)	13 518 (65.7)	18 564 (68.2)	20 701 (69.4)	18 115 (71.8)	15 403 (75.1)
Current	966 (5.2)	841 (4.1)	876 (3.2)	714 (2.4)	393 (1.6)	177 (0.9)
Former	5788 (30.9)	6191 (30.1)	7737 (28.4)	8371 (28.1)	6716 (26.6)	4913 (23.9)
Unknown or missing	10 (<0.1)	17 (<0.1)	28 (0.1)	22 (<0.1)	18 (<0.1)	26 (0.1)

^a^ Other race and ethnic category included American Indian, Alaska Native, and a write-in race option.

### Associations of Individual SDoH and ACS Guideline Scores

After adjusting for age, sex, and energy intake (model 1), all main exposures were statistically significantly and independently associated with ACS Guideline Scores (Table 2). Race and ethnicity were associated with ACS Guideline Scores, with participants identifying as Asian, Native Hawaiian, or Pacific Islander having almost twice the odds of a higher ACS Guideline Score compared with participants who identified as White (odds ratio [OR], 1.99; 95% CI, 1.86-2.14). Black participants had 32% lower odds (OR, 0.68; 95% CI, 0.64-0.72) and Latino participants had 6% lower odds (OR, 0.94; 95% CI, 0.90-0.98) of a higher ACS Guideline Score. Lower income was associated with significantly reduced odds of adherence, with participants earning less than $50 000 having 51% lower odds (OR, 0.49; 95% CI, 0.47-0.50) of a high ACS Guideline Score compared with those earning $125 000 or more. Similar trends were observed for education, as individuals with a high school education or less had 51% lower odds of a high ACS Guideline Score (OR, 0.49; 95% CI, 0.47-0.51), while those with a graduate degree had 33% higher odds (OR, 1.33; 95% CI, 1.30-1.36) compared with college graduates. Living in nonmetropolitan areas, residing in a food desert, and SHS exposure of any kind were also independently associated with lower ACS Guideline Scores. Compared with working full-time, working part-time (OR, 1.62; 95% CI, 1.57-1.66) or being retired (OR, 1.26; 95% CI, 1.22-1.30) was associated with a higher score (Table 2).

**Table 2.** Social Determinants of Health Factors Associated With ACS Guideline Adherence^a^

Characteristic	Participants, No.	Model 1^b^		Model 2^c^	
		OR (95% CI)	*P* value	OR (95% CI)	*P* value
Sex					
Female	111 694	1 [Reference]	NA	1 [Reference]	NA
Male	30 391	0.94 (0.96-1.01)	.20	0.94 (0.92-0.97)	<.001
Race and ethnicity					
Asian, Native Hawaiian, or Pacific Islander	2346	1.99 (1.86-2.14)	<.001	1.69 (1.57-1.81)	<.001
Black	3232	0.68 (0.64-0.72)	<.001	0.73 (0.69-0.78)	<.001
Latino	7767	0.94 (0.90-0.98)	.003	1.00 (0.96-1.04)	.84
White	126 788	1 [Reference]	NA	1 [Reference]	NA
Other^d^	621	0.89 (0.82-0.96)	.002	0.98 (0.91-1.06)	.68
Annual household income, $					
<50 000	21 718	0.49 (0.47-0.50)	<.001	0.66 (0.63-0.68)	<.001
50 000 to <75 000	25 023	0.59 (0.58-0.61)	<.001	0.75 (0.73-0.77)	<.001
75 000 to <100 000	24 958	0.66 (0.65-0.68)	<.001	0.79 (0.77-0.81)	<.001
100 000 to <125 000	22 797	0.74 (0.72-0.77)	<.001	0.83 (0.81-0.86)	<.001
≥125 000	45 277	1 [Reference]	NA	1 [Reference]	NA
Marital status					
Married or living with partner	108 360	1 [Reference]	NA	1 [Reference]	NA
Never married	10 707	0.81 (0.78-0.83)	<.001	0.96 (0.92-1.00)	.03
Divorced, separated, or widowed	21 258	0.84 (0.81-0.86)	<.001	1.07 (1.04-1.11)	<.001
Education level					
College graduate	49 444	1 [Reference]	NA	1 [Reference]	NA
High school or less	10 163	0.49 (0.47-0.51)	<.001	0.59 (0.56-0.61)	<.001
Some college or 2-y degree	38 017	0.61 (0.60-0.63)	<.001	0.69 (0.67-0.71)	<.001
Graduate degree	43 998	1.33 (1.30-1.36)	<.001	1.27 (1.24-1.30)	<.001
Rural urban commuting area					
Metropolitan	115 468	1 [Reference]	NA	1 [Reference]	NA
Micropolitan	11 944	0.72 (0.69-0.74)	<.001	0.85 (0.82-0.88)	<.001
Small town	3676	0.68 (0.65-0.72)	<.001	0.84 (0.79-0.89)	<.001
Rural	2244	0.64 (0.59-0.68)	<.001	0.77 (0.72-0.83)	<.001
Residing in a food desert					
No	123 997	1 [Reference]	NA	1 [Reference]	NA
Yes	9310	0.72 (0.69-0.75)	<.001	0.88 (0.84-0.91)	<.001
Secondhand smoke exposure					
Never	94 736	1 [Reference]	NA	1 [Reference]	NA
Any	46 685	0.61 (0.60-0.62)	<.001	0.70 (0.69-0.72)	<.001
Work status					
Full time	96 421	1 [Reference]	NA	1 [Reference]	NA
Part time	16 692	1.62 (1.57-1.66)	<.001	1.62 (1.57-1.67)	<.001
Retired	16 783	1.26 (1.22-1.30)	<.001	1.32 (1.28-1.36)	<.001
Other	9504	1.17 (1.13-1.22)	<.001	1.27 (1.23-1.32)	<.001

Abbreviations: ACS, American Cancer Society; NA, not applicable; OR, odds ratio.

^a^ Total ACS Guideline Score ranged from 0 to 8, with higher scores indicating better adherence to ACS Guidelines for Cancer Prevention. Reference group is  0 to 2, indicating low ACS Guideline Scores; all results describe the odds of having a higher score over the lowest score.

^b^ Adjusted for age, sex, and energy intake.

^c^ Adjusted for age, sex, energy intake, and mutually adjusted for all main exposures.

^d^ Other race and ethnic category included American Indian, Alaska Native, and a write-in race option.

After further adjustment for all main exposures (model 2), the associations among participants identifying as Latino and those never married were attenuated toward the null, although 95% CIs included the null for Latino participants only (Table 2). Additionally, the odds of a higher ACS Guideline Score among participants identifying as divorced, separated, or widowed went from being 16% lower in model 1 (OR, 0.84; 95% CI, 0.81-0.86) to 7% higher in model 2 (OR, 1.07; 95% CI, 1.04-1.11) compared with those married or living with a partner. Aside from noted exceptions, associations between SDoH and ACS Guideline Scores were minimally attenuated after mutually adjusting for all other main exposures in model 2 (Table 2).

### Joint Associations of Intersecting SDoH and ACS Guideline Score

**Figure 1.**
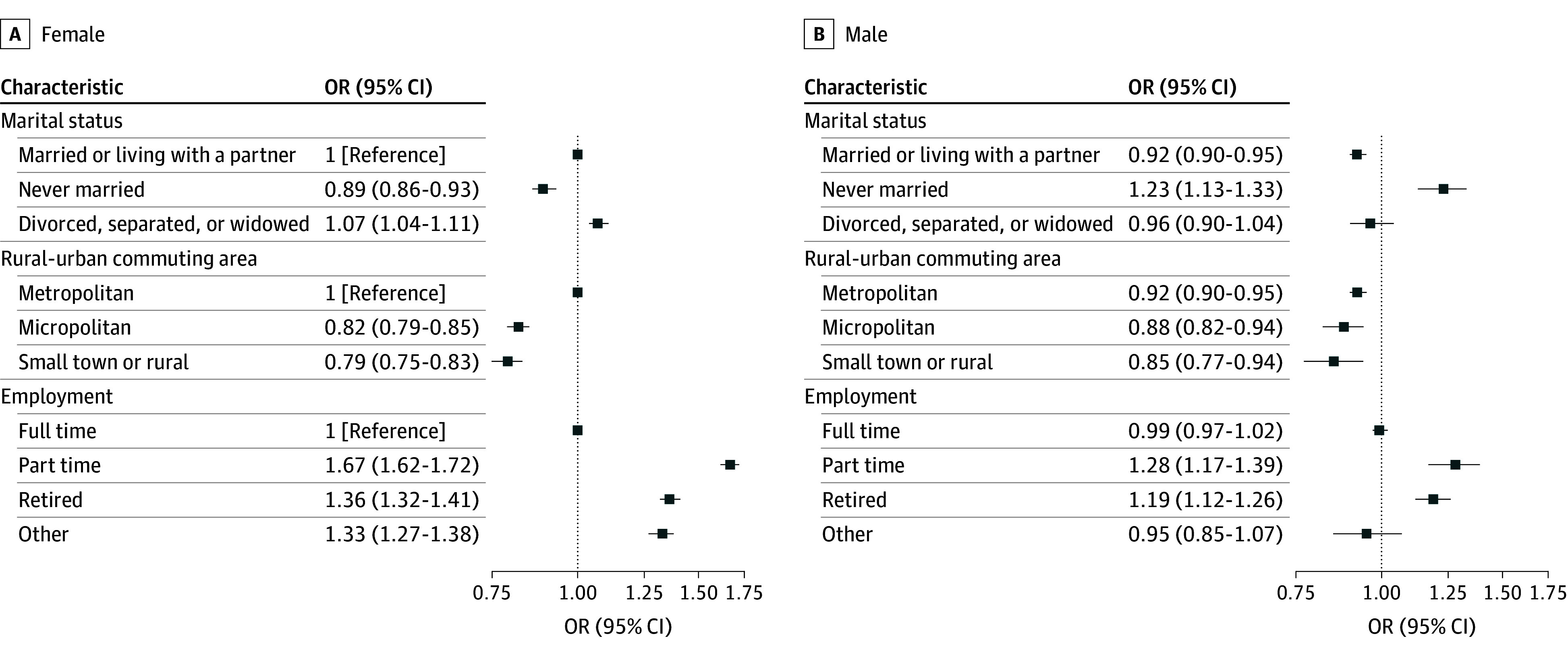
Joint Associations of Marital Status, Rurality, and Work Status by Sex OR indicates odds ratio.

**Figure 2.**
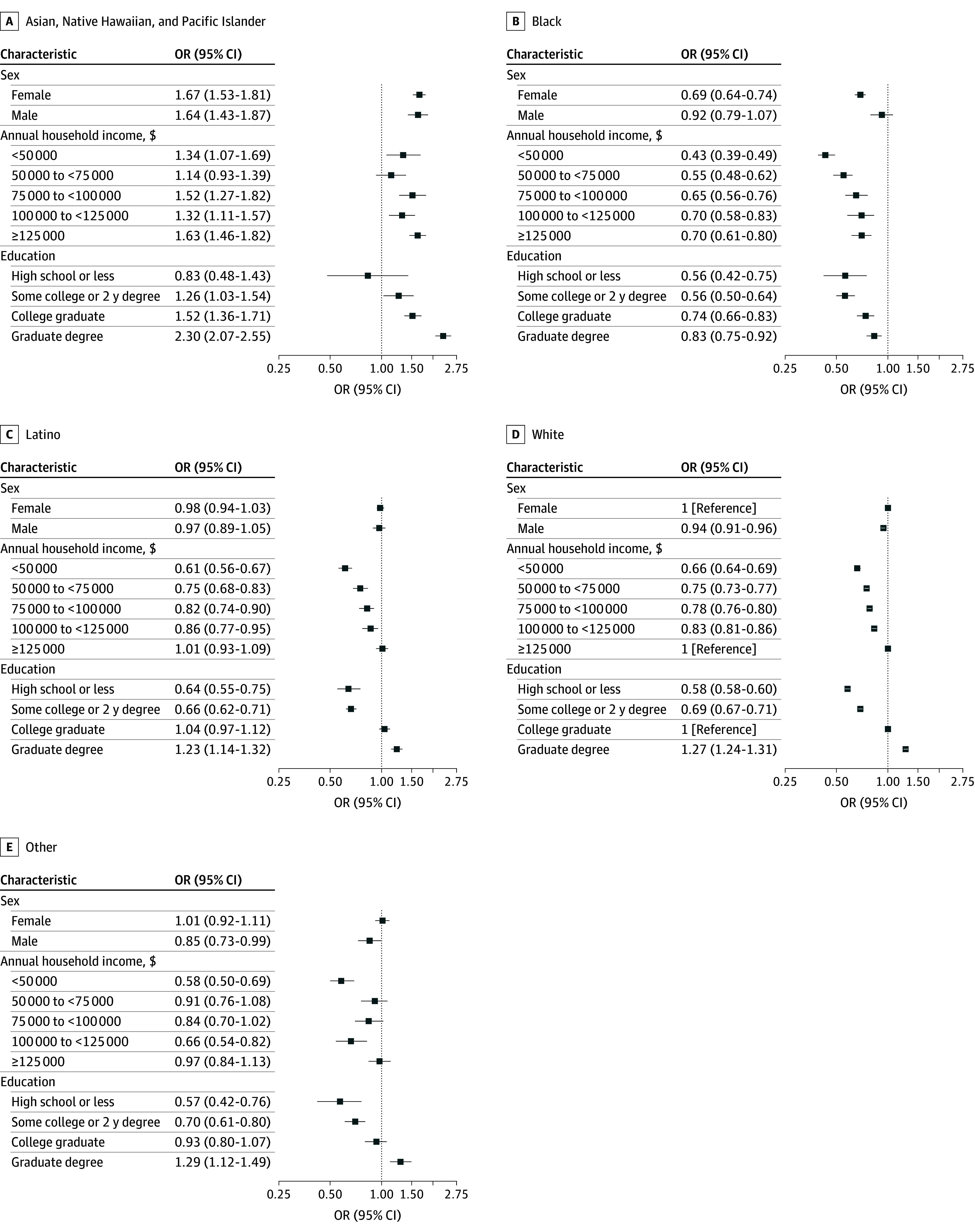
Joint Associations of Sex, Educational Attainment, and Income Level by Race and Ethnicity OR indicates odds ratio. Other race and ethnic category included American Indian, Alaska Native, and a write-in race option.

There were notable sex differences in the association between marital status and ACS Guideline Scores, particularly among those who never married, where men were 1.23 times more likely (OR, 1.23; 95% CI, 1.13-1.33), and women were 11% less likely (OR, 0.89; 95% CI, 0.86-0.93), to have a high ACS Guideline Score compared with women married or living with a partner (Figure 1; eTable 4 in Supplement 1). Female metropolitan participants had higher ACS Guideline Scores compared with all other sex and residential group combinations (Figure 1; eTable 4 in Supplement 1). Joint associations by sex and race and/or ethnicity suggested Black female participants had 31% lower odds of a high ACS Guideline Score compared with White female individuals (OR, 0.69; 95% CI, 0.64-0.74) (Figure 2; eTable 3 in Supplement 1).

**Figure 3.**
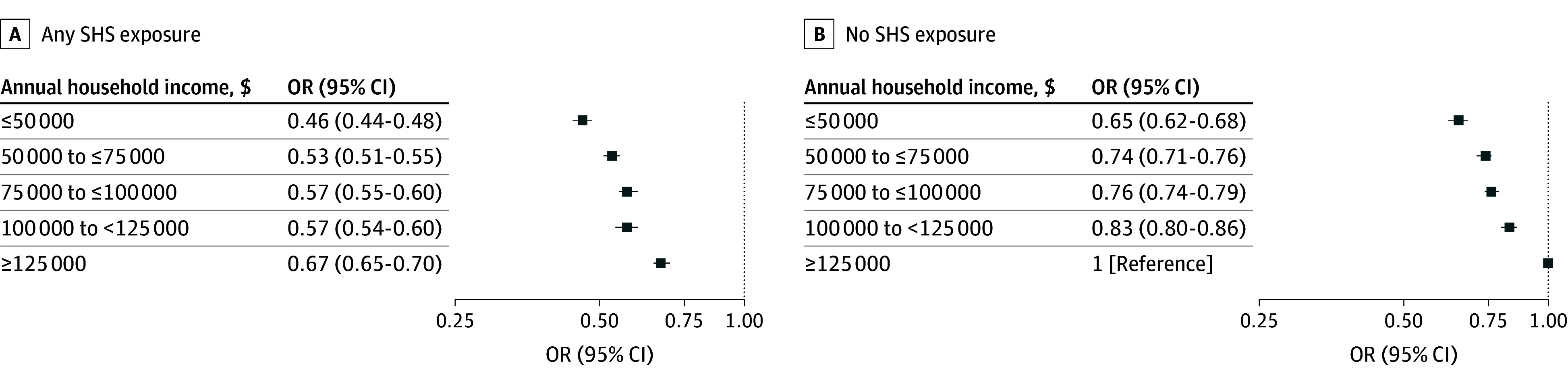
Association **B**etween Secondhand Smoke (SHS) Exposure and American Cancer Society Guideline Scores by Income Level OR indicates odds ratio.

Higher income and education levels were individually associated with greater ACS Guideline Scores; however, these associations were not consistently observed across all racial and ethnic groups. Across all education and income levels, Black participants had significantly lower odds of a high ACS Guideline Score compared with White participants (Figure 2; eTable 3 in Supplement 1). Asian, Native Hawaiian, or Pacific Islander participants earning $125 000 or more had 63% higher odds of a high ACS Guideline Score compared with White participants in the same income bracket (OR, 1.63; 95% CI, 1.46-1.82). Regardless of race and ethnicity, participants earning lower incomes had lower scores (Figure 2; eTable 3 in Supplement 1). Adherence decreased as income lowered across participants with and without SHS exposure compared with individuals earning at least $125 000 with no SHS exposure (Figure 3; eTable 5 in Supplement 1).

## Discussion

In this cross-sectional study, race and ethnicity, income, marital status, educational attainment, rural vs urban residence, living in a food desert, work status, and SHS exposure were all independently associated with co-occurring health behaviors measured by the ACS Guideline Score for Diet and Physical Activity for Cancer Prevention. Lower socioeconomic status (ie, income and education), SHS exposure, and rural residence were associated with lower scores. Being retired, having higher education, or identifying as Asian, Native Hawaiian, or Pacific Islander were associated with higher ACS Guideline Scores.

Previous research indicates that financial and educational barriers (ie, economic stability) can impede access to health-promoting resources and behaviors.^4^ Collectively, engaging in individual and co-occurring health behaviors, such as those measured by the ACS Guideline Score, was associated with higher socioeconomic status. Racial disparities among Black participants were the most prominent, in which Black women experienced 31% lower odds of a high ACS Guideline Score (ie, 7-8) compared with White women. Furthermore, Black participants with advanced degrees were less likely to have a high ACS Guideline Score than their highly educated White counterparts suggesting that the associations with educational attainment did not apply equally to Black and White participants. The lack of protective associations of higher socioeconomic status among certain racial groups, particularly Black individuals, may reflect systemic inequities and cultural differences in health behaviors.^19,20,21^ The findings of this study identified several groups at high risk of poor adherence to the ACS Guideline (ie, low ACS Guideline Score, 0-2), highlighting individuals most vulnerable to poor co-occurring health behaviors.

Joint associations between marital status and sex are worth noting. Never married men were 1.23 times more likely to have a higher ACS Guideline Score than married women; whereas, never married women had 11% lower odds. These findings are not consistent with existing literature, as most research links marriage with numerous positive physical and mental health outcomes, especially among men. For example, several large studies have found that being unmarried is associated with a higher risk of cancer,^22,23^ premature mortality,^22^ mental disorders,^24^ high blood pressure,^24,25^ and substance use disorders^26^ compared with being married,^25^ and associations are generally most impactful in men. Social scientists have attributed these protective associations to a variety of reasons including selection effect: marriage is more likely to occur among happier and healthier individuals.^27^ Other reasons include improved economic well-being associated with being partnered, leading to increased access to health care and reduced financial stress,^28^ and increased social connection leading to emotional fulfillment and the promotion of behaviors such as eating healthy and regular exercise.^29,30^ CPS-3 participants reported being more adherent in some health behaviors (eg, physical activity) than the US population; thus, it is possible these findings may not be generalizable to other groups. Our results illuminate underlying complexities associated with the influence of marital status and sex on health behaviors, warranting further investigation.

Health and well-being are significantly impacted by our environments.^31^ In this study, individuals residing in nonmetropolitan areas and food deserts had lower ACS Guideline Scores. This may be because metropolitan areas provide more opportunities to engage in healthy behaviors, including safer built environments for PA and access to healthier foods.^16^ Additionally, we saw that, across all income levels, individuals who were exposed to SHS had lower odds of a high ACS Guideline Score compared with those never exposed to SHS with gross household incomes of $125 000 or more. The impact of a lower income on ACS Guideline Scores among participants never experiencing SHS exposure was clearer than the association among those with SHS exposure. Similarly, our findings suggest that even a high income level does not fully offset the risk associated with SHS exposure, serving as a proxy for persistent and established disparities in SHS exposure.^32,33^ Individuals with household incomes below 130% of the Federal Poverty Level are more frequently exposed to SHS than those with household incomes exceeding 350% of the Federal Poverty Level.^34^

Adherence to co-occurring health behaviors as captured by the ACS Guideline Score is influenced by SDoH. These findings underscore the need for targeted health policies aimed at promoting health equity and reducing disparities. Education remains a foundational tool for addressing SDoH with the largest impact on public health.^35,36^ Therefore, practitioners should actively educate and encourage adherence to evidence-based recommendations, such as the ACS Guideline for Cancer Prevention, particularly among individuals at risk. To be effective, such efforts must account for the full spectrum of SDoH influencing individual health status, ensuring that interventions are tailored to address and eliminate barriers. Future research should explore how regional policies and political climates intersect with SDoH to influence adherence to cancer prevention guidelines. This could help identify structural factors contributing to disparities and inform targeted interventions.

### Limitations

This study has limitations that should be mentioned. Although CPS-3 is diverse in terms of geography, the proportion of White and female participants was higher than the numbers of men and participants representing other racial and/or ethnic groups; therefore, the findings of this study may not be generalizable to the US population. However, the large sample size of CPS-3 allowed for very precise estimates, even within less populous subgroups. Additionally, this study relied on self-reported diet, physical activity, and weight data, which may be subject to recall or social desirability bias; however, the physical activity,^37^ diet,^15^ and weight^38^ survey items used within CPS-3 have all been found to be valid against relevant criterion. Such interpretations may still be subject to bias toward the null. Furthermore, the cross-sectional design inhibits our ability to infer causality or directionality. Like previous studies,^11,39^ we collapsed the tail distributions of the ACS Guideline Score (eg, 0-2 and 7-8) due to sparse numbers; this decision may reduce interpretability and variation, particularly in understanding gradients of adherence. Future research should consider exploring the full 0-8 scale to address this limitation. While the World Health Organization and Healthy People domains for SDoH guided the selection of social, economic, and geographical factors measured in this study, we were unable to include other potentially important factors, such as health insurance status. Although the ACS Guideline for Cancer Prevention changed minimally from 2012 to 2020, we acknowledge the slight discordance of analyzing adherence to the 2020 update in a population from 2015.

## Conclusions

This cross-sectional study found various SDoH associated with co-occurring health behaviors, identifying Black female participants, individuals living in food deserts, and participants with SHS exposure as most vulnerable for poor health behaviors. Our findings emphasize the need to examine patterns that may determine simultaneous lifestyle behaviors and drive health disparities in the US. Additional research is needed to identify behavioral interventions for vulnerable subgroup populations, focusing on co-occurring health behaviors, rather than individual ones.

## Notes

Supplement 1. eTable 1. 2020 American Cancer Society (ACS) Cancer Prevention Guideline Scoring eTable 2. 2020 American Cancer Society Cancer Prevention Guideline Diet Score eTable 3. Social determinant of health factors associated with ACS Guideline Scores by race and ethnicity eTable 4. Social determinant of health factors associated with ACS Guideline Scores by sex eTable 5. Association of secondhand smoke (SHS) exposure and ACS Guideline Scores by income

Supplement 2. Data Sharing Statement
