# Supplementary material for: Social Determinants of Health and Cancer Prevention Guideline Behaviors
Source: JAMA Netw Open. 2025 Nov 7;8(11):e2542330. doi: 10.1001/jamanetworkopen.2025.42330 (PMC12595537; doi:10.1001/jamanetworkopen.2025.42330)
Supplement: Supplement 1. — eTable 1. 2020 American Cancer Society (ACS) Cancer Prevention Guideline Scoring eTable 2. 2020 American Cancer Society Cancer Prevention Guideline Diet Score eTable 3. Social determinant of health factors associated with ACS Guideline Scores by race and ethnicity eTable 4. Social determinant of health factors associated with ACS Guideline Scores by sex eTable 5. Association of secondhand smoke (SHS) exposure and ACS Guideline Scores by income [file jamanetwopen-e2542330-s001.pdf]

# Supplemental Online Content

Chiang KE, Padilla HM, Callands T, et al. Social determinants of health associated with cancer prevention guideline behaviors. *JAMA Netw Open.* 2025;8(11):e2542330. doi:10.1001/jamanetworkopen.2025.42330

**eTable 1.** 2020 American Cancer Society (ACS) Cancer Prevention Guideline Scoring

**eTable 2.** 2020 American Cancer Society Cancer Prevention Guideline Diet Score

**eTable 3.** Social determinant of health factors associated with ACS Guideline Scores by race and ethnicity

**eTable 4.** Social determinant of health factors associated with ACS Guideline Scores by sex

**eTable 5.** Association of second-hand smoke (SHS) exposure and ACS Guideline Scores by income

This supplemental material has been provided by the authors to give readers additional information about their work.

© 2025 Chiang KE et al. *JAMA Network Open.*

**eTable 1. 2020 American Cancer Society (ACS) Cancer Prevention Guideline Scoring**

| ACS Cancer Prevention Guidelines | Lifestyle Component | Categorization | Score |
|---|---|---|---|
| Achieve and maintain a healthy body weight throughout life. | **Body mass index (BMI)**, *kg/m²* | ≥30 at any time point | 0 |
| | | Other combinations | 1 |
| | | 18.5 - < 25 at both time points | 2 |
| Engage in 150-to-300 minutes of moderate-to-vigorous physical activity per week, or 75-to-150 minutes of vigorous-intensity physical activity, or an equivalent combination achieving or exceeding the upper limit of 300 minutes per week is optimal. | **Physical activity**, *MET-h/wk* | < 7.5 | 0 |
| | | 7.5 - < 15 | 1 |
| | | 15+ | 2 |
| Limit their consumption to no more than 1 drink per day for women and 2 drinks per day for men. | **Alcohol intake**, *drinks/day* | > 1 *(women)*; > 2 *(men)* | 0 |
| Follow a healthy dietary eating pattern that includes a variety of vegetables, fruits, and whole grains; limits red and processed meats, sugar-sweetened beverages and highly processed foods and refined grain products. | **Diet score** | 1st tertile (< 4) | 0 |
| | | 2nd tertile (4 - <8) | 1 |
| | | 3rd tertile (8-12) | 2 |
| | | **Total ACS Guideline Score:** | **0-8** |

*Scores are weighted equally on a low to high scale, with higher scores indicating better health behaviors/ACS Guideline adherence and low scores indicating poor ACS Guideline adherence/health behaviors.*

© 2025 Chiang KE et al. *JAMA Network Open*.

**eTable 2. 2020 American Cancer Society Cancer Prevention Guideline Diet Score**

| Component | Dietary Constituents | Units | Min Score | Max Score | Scoring (sex-specific quartiles) |
|---|---|---|---|---|---|
| **Adequacy** | | | | | |
| Vegetables | Vegetable intake (excludes starchy vegs like potatoes and corn) | svgs/d, unit=servings/day (0.5 c of veg; 1 cup of green leafy (1 cup = 236.59 g) | 0 | 0.75 | Q1= 0<br>Q2= 0.25<br>Q3= 0.5<br>Q4=0.75 |
| | Unique Vegetable Variety | # line items | 0 | 0.75 | Q1=0<br>Q2=0.25<br>Q3=0.5<br>Q4=0.75 |
| Fruits | Fruit intake (excluding fruit juices) | svgs/d, unit=servings/day (0.5 c of berries; 1 cup other fruits=236.59 g; 1 med fruit; 0.5 medium avocado) | 0 | 0.75 | Q1= 0<br>Q2= 0.25<br>Q3= 0.5<br>Q4=0.75 |
| | Unique Fruit Variety | # line items | 0 | 0.75 | Q1=0<br>Q2=0.25<br>Q3=0.5<br>Q4=0.75 |
| Whole Grains | Whole grains | svgs/d, unit=grams/day | 0 | 3 | Q1= 0<br>Q2= 1<br>Q3= 2<br>Q4= 3 |
| Red and processed meat | Red and processed meat | svgs/d, unit=servings/day; 1 srv= 4 oz. unprocessed meat; 1.5 oz. processed meat (1 oz. = 28.35 g) | 0 | 3 | Q1= 3<br>Q2= 2<br>Q3= 1<br>Q4= 0 |

© 2025 Chiang KE et al. *JAMA Network Open*.

| | | | | | |
|---|---|---|---|---|---|
| HPF, Refined grains | Highly processed foods, refined grains | Ratio of calories from these to total calories | 0 | 1.5 | Q1=1.5<br>Q2=1<br>Q3=0.5<br>Q4=0 |
| SSB | Sugar-sweetened beverages (does not include fruit juices) | svgs/d, unit=servings/day = 1 ser= 8oz (1 oz. = 28.35 g) | 0 | 1.5 | Never (e.g., non-drinkers): <= 0 svgs/d, these people should get 1.5 pts<br>< 3 days per week (<3/7): 1pt<br>>=3 to <7 days per week (>=3/7 & <1): 0.5 pts<br>Daily or more: 7 days per week (>=1): 0 pts |
| | | Total possible score: | 0 | 12 | |

© 2025 Chiang KE et al. *JAMA Network Open*.

**eTable 3. Social determinant of health factors associated with ACS Guideline Scores by race and ethnicity[a]**

| | Race and Ethnicity | | | | | | | | | |
|---|---|---|---|---|---|---|---|---|---|---|
| | Asian, Native Hawaiian, or Pacific Islander | | Black | | Latino | | White | | Other | |
| | n | OR (95%CI) | n | OR (95%CI) | n | OR (95%CI) | n | OR (95%CI) | n | OR (95%CI) |
| **Sex[b]** | | | | | | | | | | |
| Female | 1,681 | 1.67 (1.53-1.81) | 2,712 | 0.69 (0.64-0.74) | 6,020 | 0.98 (0.94-1.03) | 99,785 | **1 [Reference]** | 1,496 | 1.01 (0.92-1.11) |
| Male | 665 | 1.64 (1.43-1.87) | 520 | 0.92 (0.79-1.07) | 1,747 | 0.97 (0.89-1.05) | 27,003 | 0.94 (0.91-0.96) | 456 | 0.85 (0.73-0.99) |
| **Income, $[c]** | | | | | | | | | | |
| <50,000 | 234 | 1.34 (1.07-1.69) | 925 | 0.43 (0.39-0.49) | 1,622 | 0.61 (0.56-0.67) | 18,465 | 0.66 (0.64-0.69) | 472 | 0.58 (0.50-0.69) |
| 50,000 to <75,000 | 287 | 1.14 (0.93-1.39) | 732 | 0.55 (0.48-0.62) | 1,418 | 0.75 (0.68-0.83) | 22,222 | 0.75 (0.73-0.77) | 364 | 0.91 (0.76-1.08) |
| 75,000 to <100,000 | 371 | 1.52 (1.27-1.82) | 522 | 0.65 (0.56-0.76) | 1,315 | 0.82 (0.74-0.90) | 22,415 | 0.78 (0.76-0.80) | 335 | 0.84 (0.70-1.02) |
| 100,000 to <125,000 | 396 | 1.32 (1.11-1.57) | 391 | 0.70 (0.58-0.83) | 1,161 | 0.86 (0.77-0.95) | 20,597 | 0.83 (0.81-0.86) | 252 | 0.66 (0.54-0.82) |
| 125,000 or more | 1,032 | 1.63 (1.46-1.82) | 620 | 0.70 (0.61-0.80) | 2,153 | 1.01 (0.93-1.09) | 40,976 | **1 [Reference]** | 496 | 0.97 (0.84-1.13) |
| **Education level[d]** | | | | | | | | | | |
| High school or less | 40 | 0.83 (0.48-1.43) | 142 | 0.56 (0.42-0.75) | 557 | 0.64 (0.55-0.75) | 9,293 | 0.58 (0.56-0.60) | 131 | 0.57 (0.42-0.76) |
| Some college or 2-y degree | 305 | 1.26 (1.03-1.54) | 818 | 0.56 (0.50-0.64) | 2,292 | 0.66 (0.62-0.71) | 33,937 | 0.69 (0.67-0.71) | 665 | 0.70 (0.61-0.80) |
| College graduate | 859 | 1.53 (1.36-1.71) | 1,030 | 0.74 (0.66-0.83) | 2,513 | 1.04 (0.97-1.12) | 44,460 | **1 [Reference]** | 582 | 0.93 (0.80-1.07) |
| Graduate degree | 1,137 | 2.30 (2.07-2.55) | 1,225 | 0.83 (0.75-0.92) | 2,357 | 1.23 (1.14-1.32) | 38,714 | 1.27 (1.24-1.31) | 565 | 1.29 (1.12-1.49) |

[a]For exposures with statistically significant interactions, p-int <.001. ACS Guideline Score is on a 0-8 scale, with higher scores indicating better scores/health behaviors. Reference group for ACS Guideline Score was 0-2, indicating low ACS Guideline Scores (i.e., low adherence).
[b]Model included age, energy intake, race/ethnicity, income, marital status, education, RUCA, residence in a food desert, second-hand smoke exposure, work status, and a race*sex joint variable.
[c]Model included age, sex, energy intake, marital status, education, RUCA code, residence in a food desert, second-hand smoke exposure, work status, and a race*income joint variable.
[d]Model included age, sex, energy intake, income, marital status, RUCA code, residence in a food desert, second-hand smoke exposure, work status, and a race*education joint variable.
Note: 'Other' race and ethnic category included American Indian, Alaskan Native, and a write-in race option.

© 2025 Chiang KE et al. *JAMA Network Open*.

**eTable 4. Social determinant of health factors associated with ACS Guideline Scores by sex[a]**

| | Female | | | Male | | |
|---|---|---|---|---|---|---|
| **Characteristic** | **n** | **OR (95% CI)** | **p-value** | **n** | **OR (95% CI)** | **p-value** |
| **Marital status[b]** | | | | | | |
| Married or living with partner | 83,601 | **1 [Reference]** | | 24,759 | 0.92 (0.90-0.95) | <.001 |
| Never married | 8,919 | 0.89 (0.86-0.93) | <.001 | 1,788 | 1.23 (1.13-1.33) | <.001 |
| Divorced, separated, or widowed | 18,926 | 1.07 (1.04-1.11) | <.001 | 2,332 | 0.96 (0.90-1.04) | 0.32 |
| **Rurality[c]** | | | | | | |
| Metropolitan | 90,511 | **1 [Reference]** | | 24,957 | 0.92 (0.90-0.95) | <.001 |
| Micropolitan | 9,488 | 0.82 (0.79-0.85) | <.001 | 2,456 | 0.88 (0.82-0.94) | 0.00 |
| Small town/Rural | 4,771 | 0.79 (0.75-0.83) | <.001 | 1,149 | 0.85 (0.77-0.94) | 0.001 |
| **Work status[d]** | | | | | | |
| Full time | 73,096 | **1 [Reference]** | | 23,325 | 0.99 (0.97-1.02) | 0.49 |
| Part time | 15,167 | 1.67 (1.62-1.72) | <.001 | 1,525 | 1.28 (1.17-1.39) | <.001 |
| Retired | 12,729 | 1.36 (1.32-1.41) | <.001 | 4,054 | 1.19 (1.12-1.26) | <.001 |
| Other | 8,549 | 1.33 (1.27-1.38) | <.001 | 955 | 0.95 (0.85-1.07) | 0.40 |

[a]ACS Guideline Score is on a 0-8 scale, with higher scores indicating better scores/health behaviors. Reference group for ACS Guideline Score was 0-2, indicating low ACS Guideline Scores (i.e., low adherence).

[b]Model included age, energy intake, race/ethnicity, income, education, RUCA code, residence in a food desert, work status, second-hand smoke exposure, and a joint marital status*sex variable.

[c]Model included age, energy intake, race/ethnicity, income, education, marital status, residence in a food desert, work status, second-hand smoke exposure, and a joint RUCA*sex variable.

[d]Model included age, energy intake, race/ethnicity, income, education, RUCA code, marital status, residence in a food desert, second-hand smoke exposure, and a joint work status*sex variable.

© 2025 Chiang KE et al. *JAMA Network Open*.

**eTable 5. Association of second-hand smoke (SHS) exposure and ACS Guideline Scores by income[a]**

| | Never | | Any | |
|---|---|---|---|---|
| **Income level, $[b]** | **n** | **OR (95% CI)** | **n** | **OR (95% CI)** |
| <50,000 | 12,123 | 0.65 (0.62-0.68) | 9,448 | 0.46 (0.44-0.48) |
| 50,000 to <75,000 | 15,383 | 0.74 (0.71-0.76) | 9,506 | 0.53 (0.51-0.55) |
| 75,000 to <100,000 | 16,239 | 0.76 (0.74-0.79) | 8,605 | 0.57 (0.55-0.60) |
| 100,000 to <125,000 | 15,560 | 0.83 (0.80-0.86) | 7,146 | 0.57 (0.54-0.60) |
| 125,000 or more | 33,792 | **1 [Reference]** | 11,326 | 0.67 (0.65-0.70) |

[a]For exposures with statistically significant interactions, p-int <.001. ACS Guideline Score is on a 0-8 scale, with higher scores indicating better scores/health behaviors. Reference group for ACS Guideline Score was 0-2, indicating low ACS Guideline Scores (i.e., low adherence).

[b]Model included age, sex, energy intake, income, education, RUCA code, marital status, residence in a food desert, second-hand smoke exposure, and a joint SHS*income variable.

© 2025 Chiang KE et al. *JAMA Network Open*.
