# Supplementary material for: Social Determinants of Health and Cancer Prevention Guideline Behaviors
Source: JAMA Netw Open. 2025 Nov 7;8(11):e2542330. doi: 10.1001/jamanetworkopen.2025.42330 (PMC12595537; doi:10.1001/jamanetworkopen.2025.42330)
Supplement: Supplement 2. — Data Sharing Statement [file jamanetwopen-e2542330-s002.pdf]

Data Sharing Statement

Chiang. Social Determinants of Health Associated With Cancer Prevention Guideline Behaviors. *JAMA Netw Open*. Published November 07, 2025.
doi:10.1001/jamanetworkopen.2025.42330

Data
Data available: Yes
Data types: Deidentified participant data
How to access data: Data are available from the American Cancer Society by following the ACS Data Access Procedures (https://www.cancer.org/research/population-science/research-collaboration.html) for researchers who meet the criteria for access to confidential data. Please email cohort.data@cancer.org to inquire about access.
When available: With publication

Supporting Documents
Document types: None

Additional Information
Who can access the data: Data are available from the American Cancer Society by following the ACS Data Access Procedures (https://www.cancer.org/research/population-science/research-collaboration.html) for researchers who meet the criteria for access to confidential data. Please email cohort.data@cancer.org to inquire about access.
Types of analyses: Any purpose
Mechanisms of data availability: with investigator support, after approval of a proposal, and a signed data access agreement.
